# Social and asocial learning in zebrafish are encoded by a shared brain network that is differentially modulated by local activation

**DOI:** 10.1038/s42003-023-04999-5

**Published:** 2023-06-13

**Authors:** Júlia S. Pinho, Vincent Cunliffe, Kyriacos Kareklas, Giovanni Petri, Rui F. Oliveira

**Affiliations:** 1 Integrative Behavioural Biology Lab, Instituto Gulbenkian de Ciência, Oeiras, Portugal; 2 School of Biosciences, University of Sheffield, Sheffield, UK; 3 ISI Foundation and ISI Global Science Foundation, Torino, Italy; 4 ISPA-Instituto Universitário, Lisboa, Portugal; 5 Champalimaud Neuroscience Program, Champalimaud Centre for the Unknown, Lisbon, Portugal; 6 Present Address: IMIM - Institut Hospital del Mar d′Investigacions Mèdiques, Barcelona, Spain

**Keywords:** Social behaviour, Behavioural ecology

## Abstract

Group living animals use social and asocial cues to predict the presence of reward or punishment in the environment through associative learning. The degree to which social and asocial learning share the same mechanisms is still a matter of debate. We have used a classical conditioning paradigm in zebrafish, in which a social (fish image) or an asocial (circle image) conditioned stimulus (CS) have been paired with an unconditioned stimulus (US=food), and we have used the expression of the immediate early gene *c-fos* to map the neural circuits associated with each learning type. Our results show that the learning performance is similar to social and asocial CSs. However, the brain regions activated in each learning type are distinct and a community analysis of brain network data reveals segregated functional submodules, which seem to be associated with different cognitive functions involved in the learning tasks. These results suggest that, despite localized differences in brain activity between social and asocial learning, they share a common learning module and social learning also recruits a specific social stimulus integration module. Therefore, our results support the occurrence of a common general-purpose learning module, that is differentially modulated by localized activation in social and asocial learning.

## Abstract (web summary)

Zebrafish in a classical conditioning paradigm display similar learning performance, yet distinct brain region activation, in the context of social- or asocial-conditioned stimuli, suggesting the occurrence of a general-purpose learning module that is differentially modulated by localized activation.

## Introduction

The social intelligence hypothesis^1,2^ states that living in social groups creates a demand for enhanced cognitive abilities in order to handle the variability and unpredictability of social interactions, hence driving the evolution of more complex cognitive skills (aka intelligence), and consequently selecting for larger executive brains (i.e. social brain hypothesis)^3,4^. However, two different scenarios have been proposed for how these evolved cognitive abilities implement adaptive behavior. According to a general-purpose brain scenario, mechanisms of information input, encoding, storage and retrieval are shared between functional domains (e.g. social, foraging, predator avoidance), hence, although evolved in a specific domain (e.g. social), enhanced cognitive abilities are advantageous in all domains. Alternatively, each functional domain relies on special-purpose cognitive modules, which are highly specialized with independent mechanisms of information processing. In this regard, there is an ongoing debate in the field of social cognition, on the extent to which social learning (i.e. learning from other individuals) is a general-domain or a domain-specific process^5–9^. For example, comparative studies in birds and primates show correlations between the performance on social learning and individual learning (aka asocial learning) tasks or measures of behavioral flexibility, suggesting that these traits evolved together^10–12^. Furthermore, observational learning in bumblebees has been shown to emerge through the integration of two learned associations following Pavlovian conditioning rules^13^. In contrast, there is also comparative evidence supporting the occurrence of domain-specific modules, such as the differences found in social learning, but not in individual learning, between two corvid species with differences in degree of sociality, or between human children and apes^14,15^. Moreover, intra-specific studies in mice show that maternal deprivation early in life impairs social learning whereas spatial learning is unaffected, and that communally-reared mice, when compared to single-mother reared mice, have better social competence but do not differ in spatial learning and memory capacity tests^16,17^. Finally, a third scenario has also been proposed that suggests that social learning operates on the same general learning mechanisms as asocial learning with adaptive specializations present only for the input systems (i.e. social information acquisition)^6–9^.

The study of the proximate mechanisms (i.e. genetic basis, neural circuits) of social and asocial learning can, in principle, help to clarify the occurrence of shared processes. Unfortunately, there are few studies on such mechanisms with notable exceptions for the study of observational fear learning and for social learning of food preference. In both humans and rodents, social (i.e. observational) and asocial fear conditioning share, at least partially, the same neural substrates, with the anterior cingulate cortex (ACC) processing the social information and conveying it to the amygdala, which plays then a major role in the CS-US pairing in both learning types^18–22^. In rodents, a specialized olfactory subsystem has been described that is required for the acquisition of socially transmitted food preferences^23^. Moreover, social fear learning and classic fear learning can prime each other (i.e. a prior observational fear learning will enhance fear conditioning and vice-versa; e.g. ^24^). Together these results suggest an overlap of the neural mechanisms involved in social learning and in learning from direct experience, with specializations being mainly present at the level of social information acquisition. However, the above-mentioned studies address specific brain regions that are chosen a priori as candidates for the social learning tasks, and studies using an unbiased brain network approach are lacking in this field. This is particularly important because despite the overlap of brain circuits processing social and asocial learning, the candidate brain region approach does not rule out the occurrence of specialized circuits elsewhere in the brain. Furthermore, the analysis of localized neuronal activation, which is usually the parameter studied in relation to the behavioral output, does not provide per se information on the patterns of co-activation across a brain network that may reveal either specialized or conserved modules for the two learning types. Finally, the study of the neural mechanisms of social learning has focused on mammals, and comparative data in other vertebrate species that lack evolved cortical structures is also missing.

Here we have used a classic (Pavlovian) conditioning paradigm in zebrafish, in which a social (static image of a zebrafish) or an asocial (static image of a circle) conditioned stimulus (CS) was paired with an unconditioned stimulus (US = food), to investigate the neural basis of social and asocial learning (see Supplementary Figure 1 for images of the static stimuli used). The use of a static image of a fish was used as a social stimulus since it is known that both biological motion and body shape and/or coloration pattern are cues that elicit social attraction^25,26^, and previous social learning paradigms have successfully used static social cues as CS (e.g. ^13^). The choice of this classic conditioning paradigm where the social and asocial treatments are matched for everything except the visual shape of the CS rule out putative confounding variables, such as for example the involvement of different sensory modalities in the acquisition of the social information. We have used the expression of the immediate early gene *c-fos* as a molecular marker of neuronal activity^27,28^. We have also developed a method to analyze brain network functional connectivity based on the brain regions’ co-activation matrices for each experimental treatment. In network neuroscience, such matrices are built for individual brains based on a similarity measure between the timeseries of brain parcels in fMRI, or the different channels in an EEG^29^. In the case of a molecular marker of neuronal activity, such as *c-fos*, that only provides a single snapshot per brain, the correlations of activity between brain regions (i.e. the number of positive *c-fos* cells for each pair of brain nuclei), are obtained for a set of brains from different individuals. Therefore, the estimation of the similarity in activation between regions can only be computed at the group level (i.e. using datapoints from individuals in the same treatment), effectively extracting a group-level, as opposed to individual, functional connectivity network. Finally, we used techniques from network community analysis to identify coherent sets of nodes (submodules) consistently recruited by the different cognitive tasks involved in the social and asocial learned response. It must be stressed here that learning is a multi-step process that involves encoding, storage, and retrieval of information in the brain^30^. Since we have tested the patterns of brain activation in response to the probe test (after a training phase) the identified brain areas should reflect the retrieval of the learned response, but not necessarily encoding or storage. Therefore, in this paper when we refer to brain regions or networks involved in learning we are specifically referring to those involved in the retrieval of the learned response, that may or may not also be involved in encoding or storage.

## Results

### Social and asocial classic conditioning in zebrafish

**Fig. 1:**
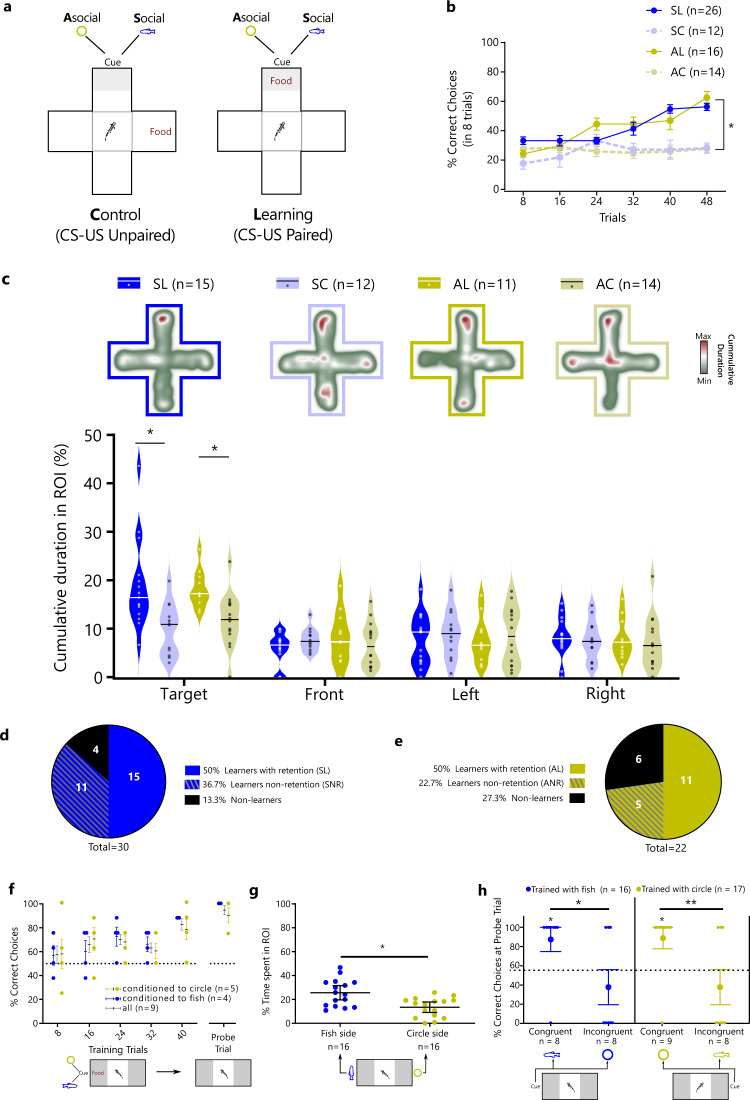
Social and asocial classic conditioning in zebrafish. **a** Schematic representation of the plus-maze paradigm: 4 groups observed a CS (social or asocial cue) paired with a US (food: bloodworms) in the same arm (paired treatments: SL and AL) hence being able to establish the CS-US association; or in different arms (unpaired treatments: SC and AC) the controls of the experiment. **b** During the training phase animals increased significantly the percentage of correct choices both in the social learning (SL, in red circles) and asocial learning (AL, in light red circles) treatments in comparison with the respective unpaired treatments [in blue circles social unpaired control (SC) and in light blue circles the asocial unpaired control (AC)]. **c** In the probe test, the cumulative duration of time spent in the RoI (heats maps provide illustrative examples of space use by representative individuals from each treatment) indicates that learners (social and asocial) increased the time spent in the target arm (line inside each violin plot indicates the median). Pie graphs indicate the proportion of learners, non-learners and non-retention animals in: **d** social; and **e** asocial conditions. **f** The ability of the animals to distinguish between the social and asocial stimuli used in this experiment was tested by conditioning the animals to approach one stimulus and avoid other, independent of their initial preference (in yellow triangles animals conditioned to approach asocial, in light pink squares individuals conditioned to approach social and in black circles the average of all individuals). **g** The preference for the social and asocial stimuli [fish (yellow circle) or circle (gray square) static 2D picture, respectively was assessed using a preference test. **h** To test social and non-social cue specificity in associative learning, animals exhibiting learning in a two-choice paradigm with local food-reward paired either with the circle or the fish shape (*p* > 50% for rewarded location) were tested at probe trials with either the cue they were trained (congruent) or the other cue (incongruent), where the significant preference for the congruent cue significantly contrasted the lack of preference for the incongruent cue [*P ≤ 0.05, ** *P* ≤ 0.01]. Asterisks indicate statistical significance at *p* < 0.05 using planned comparisons. Error bars report the standard error of the mean in the graphs of panels **b**, **f**, **g**, and **h**.

Pavlovian conditioning was assessed using a plus-maze paradigm divided into a training phase and a probe test. During the training phase, we spatially paired a social or an asocial conditioned stimulus (CS) with an unconditioned stimulus (US; food = bloodworms) in a specific location (Fig. 1a). The percentage of correct choices per session (composed of 8 trials) was measured. In the probe test (24h after the last training session), individuals only had access to the CS, and the cumulative time spent in the region of interest (RoI) of the correct arm of the maze was quantified to measure learning. Unpaired treatments were used as controls, where the CS (either social or asocial) was spatially unmatched with the US.

Animals learned both socially and asocially (learning main effect: X^2^_R_(1) = 11.39, *p* < 0.0001, η^2^_p_ = 0.079) as shown by the comparison in the percentage of correct choices between paired CS-US [social learning (SL) and asocial learning (AL)] and unpaired CS-US [social control (SC) and asocial control (AC)) treatments for social and asocial CSs (SL vs SC:X^2^_R_(1) = 6.95, *p* = 0.0089, η^2^_p_ = 0.030; AL vs AC:(X^2^_R_(1) = 28.44, *p* < 0.0001, η^2^_p_ = 0.139) (Fig. 1b). Animals in the social and asocial learning treatments (SL and AL)) acquired information at the same rate, since no significant differences between social and asocial learning curves were found either in slope (X^2^_R_(1) = 1.53, *p* = 0.22, η^2^_p_ = 0.006) or elevation (X^2^_R_(1) = 0.001, p=0.97, η^2^_p_ =0.000) (Fig. 1b). It is worth mentioning that there were also no significant differences between the social and asocial control treatments either in slope (X^2^_R_(1) = 2.0, *p* = 0.16, η^2^_p_ = 0.012) or elevation (X^2^_R_(1) = 0.14, *p* = 0.70, η^2^_p_ = 0.001) (Fig. 1b). Moreover, in control treatments, animals did not present any biased behavior towards one of the arms of the plus maze exhibiting a random proportion of choices over the trials (25% in social and asocial treatments across the training sessions).

In the probe test, individuals from the learning treatments (SL and AL) spent more time in the target arm independently if they were trained using a social (X^2^_F_(1) = 12.89, *p* = 0.001, η^2^_p_ = 0.212) or an asocial (X^2^_F_(1) = 11.53, *p* = 0.001, η^2^_p_ = 0.194) CS, when compared to the control treatments (i.e. unpaired CS-US). We did not observe any significant difference in the time spent in the other arms of the plus-maze indicating an absence of any spatial biases in the spatial use of the maze by the fish during this phase of the experiment (opposite arm to the target arm: SL vs SC,, X^2^_F_(1)=1.22, p=0.276, η^2^_p_ =0.025, AL vs AC,, X^2^_F_(1)=0.32, p=0.577, η^2^_p_ =0.007; left of the target arm: SL vs SC, X^2^_F_(1)=0.42, p=0.522, η^2^_p_ =0.009, AL vs AC, X^2^_F_(1)=0.06, p=0.801, η^2^_p_ =0.001; right of the target arm: SL vs SC, X^2^_F_(1)=0.25, p=0.617, η^2^_p_ =0.005, AL vs AC, X^2^_F_(1)=0.21, p=0.649, η^2^_p_ =0.004) (Fig. 1c). It must be stressed that only successful animals in the training phase (i.e. that acquired the learning) were tested in the test phase. Thus, this paradigm allowed the classification of individuals in the learning treatments (SL and AL) into three different categories: non-learners, learners and learners that forget the learned information from the last learning session to the probe test (i.e. no-retention group: social non-retention (SNR) and asocial non-retention (ANR)).The learners were able to acquire the information and recall it (50% of individuals in both the social and asocial learning treatments); the no-retention group were animals that despite showing a learning curve during the training sessions did not recall the acquired information in the probe test (36.67% individuals in the social group (SNR) and 22.73% individuals in the asocial group(ANR)); and a small percentage of individuals that did not improve the performance over the training sessions (13.33% individuals in the social groups and 27.27% individuals in the asocial group) were classified as non-learners (Fig. 1d, e). The non-learners and no-retention animals were identified using the lower limit of the 95% interval of confidence in the training phase (based on the values from the last day of training) and cumulative time in the RoI of the target arm during the probe test as criteria, respectively. The proportion of learners (X^2^(1) = 0, *p* = 1, OR = 1), non-learners (X^2^(1) = 1.56, *p* = 0.21, OR = 0.5) and non-retention (X^2^(1) = 1.14, *p* = 0.29, OR = 1.58) individuals did not differ between social and asocial learning treatments (Fig. 1d, e).

Given the lack of difference in behavioral measures between social and asocial learning it was important to make sure that the individuals can discriminate the two CS stimuli used in this test. Thus, a visual discrimination task was used to assess if zebrafish can discriminate between the two stimuli (social and asocial CS), where one stimulus (either the social or the asocial CS) was associated with a reward (food) and the other with a punishment (netting), in a balanced manner; that is, in half the animals the social stimulus was associated with reward and in the other half it was associated with punishment. In the probe test, only the CSs were presented, and, if individuals were able to discriminate between the two stimuli, they should prefer the arm associated with reward. This test indicated that zebrafish could distinguish between the social and asocial stimuli used in this study and that the learning curve for the acquisition of these discrimination was similar when either the social or the asocial were paired with the reward (slope X^2^_R_(1) = 1.74, *p* = 0.22, η^2^_p_ = 0.178; elevation X^2^_R_(1) = 0.43, *p* = 0.53, η^2^_p_ = 0.046; Fig. 1f). Given that social animals usually have an innate preference for social cues (e.g. refs. ^31–33^) we have also tested the preference of zebrafish for the social stimuli used here to make sure that it had a positive valence. Preference was assessed using a choice test, where animals could choose between spending time near the social vs. the asocial stimuli used in our study. As predicted, a preference for the social stimulus was observed (t(15) = 2.55, *p* = 0.02, η^2^_p_ = 0.302; Fig. 1g). Finally, to make sure that the used social and asocial stimuli constitute separate perceptual inputs into the learning mechanism, in the sense that generalization from one cue to the other does not occur, animals trained with one cue in the training phase were tested with the other cue in the test phase (e.g. individuals trained with social cue and tested with asocial cue, and vice-versa). Thus, following training, animals that learned, from either the circle or the fish-shape learning groups, were tested with either a congruent cue (circle-trained tested with the circle or fish-trained tested with the fish) or an incongruent cue (circle-trained tested with or fish-trained tested with the circle). Thus, the specificity of each cue in associative learning, as opposed to potential generalization across cues, was tested by comparing success rates between congruent and incongruent cue tests for both circle-trained and fish-trained animals. Animals that were tested at probe trials with a congruent cue exhibited greater success rates than those tested with an incongruent cue (Fig. 1h), both for the circle-trained (*Z*_*16*_ = 2.56, *P* = 0.010, Δp̂ = 0.514, OR = 9.12) and fish-trained (*Z*_*15*_ = 2.41, *P* = 0.016, Δp̂ = 0.500, OR = 10.36) group. Also, being tested with a congruent cue elicited higher than chance success rates (circle-trained: p̂ = 0.889, df = 9, *Z* = 2.33, *P* = 0.020, OR = 6.62; fish-trained: p̂ = 0.875, df = 8, *Z* = 2.12, *P* = 0.034, OR = 5.82), as opposed to being tested with an incongruent cue (circle-trained: p̂ = 0.375, df = 8, *Z* = 0.71, *P* = 0.480, OR = 1.60; fish-trained: p̂ = 0.375, df = 8, *Z* = 0.71, *P* = 0.480, OR = 1.60). Together, these results indicate a lack of generalization across cues in this learning paradigm supporting the view that social and asocial CS are triggering different perceptual mechanisms.

In summary, in zebrafish both social and asocial cues are equally efficient as a CS in a classic conditioning paradigm, despite zebrafish having an innate preference for the social cue, and generalization across cues does not occur.

### Brain regions associated with social and asocial classic conditioning in zebrafish

**Fig. 2:**
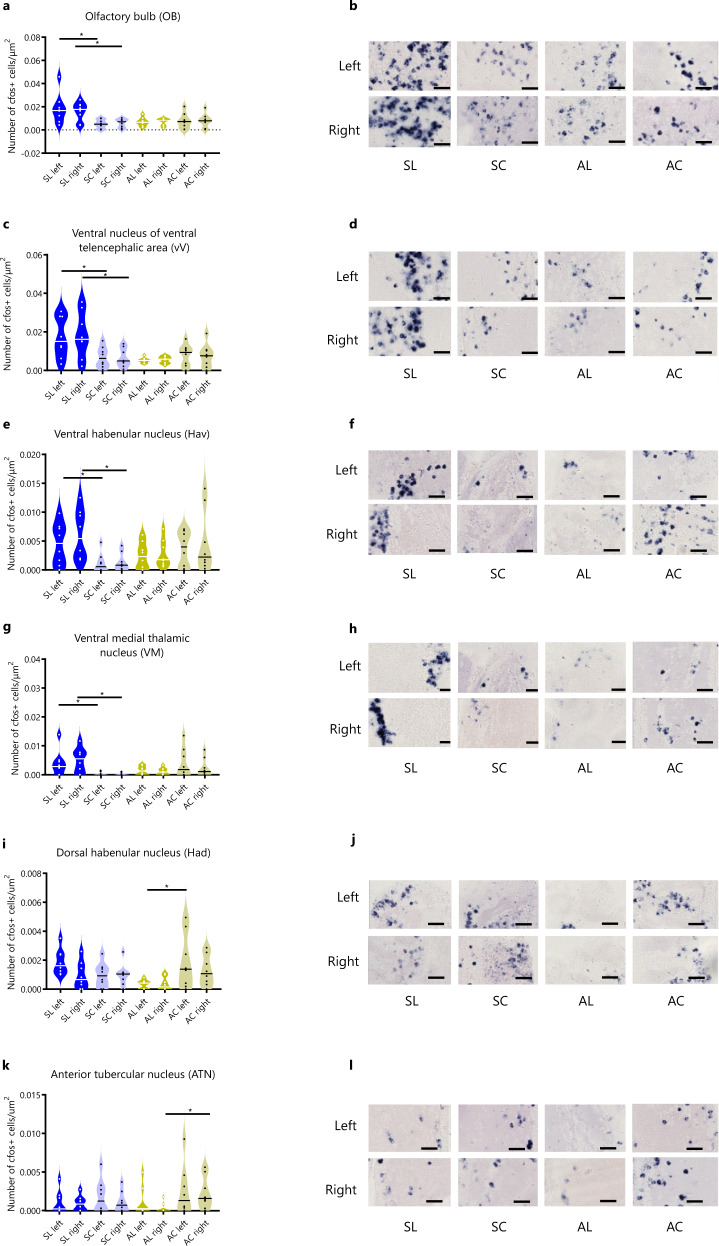
Neuronal activity associated with social (a–h) and asocial (i–l) classic conditioning in zebrafish assessed by in situ hybridization of the immediate early gene *c-fos*. Representative photomicrographs of *c-fos* in situ hybridization in areas that present significant differences associated with learning: OB **b**, Vv **d**, Hav **f**, VM **h**, Had **j**, and ATN **l**. Asterisks indicate statistical significance at *p* < 0.05 using planned comparisons followed by Benjamini and Hochberg’s method for multiple comparisons *p*-value adjustment. The line within each violin plot indicates the median. Scale bars represent 40 µm. See Table 1 for abbreviations of brain regions.

The brain regions (see Table 1 for list of regions studied and their abbreviations) associated with social and asocial learning were determined using the expression of the immediate early gene *c-fos*, a marker of neuronal activation, by in situ hybridization. For this purpose, 8 representative animals from each group (i.e. with their behavior score closer to the mean of their experimental treatment) have been selected. Because of possible laterality effects the expression of *c-fos* was measured on both brain hemispheres (noted below as left or right for each brain region), and cell counts on each hemisphere of the same brain region have been analyzed separately. We identified as brain nuclei involved in social (SL) or asocial (AL) learned responses those that presented significant differences in *c-fos* positive cells between animals of the paired treatments (SL or AL) that were able to acquire and recall the CS and the respective unpaired control treatments (SC or AC, respectively). The following areas showed increased activation associated with social learning: olfactory bulb (OB) (left: X^2^_F_(1) = 8.87, *p* = 0.022, η^2^_p_ = 0.397; right: X^2^_F_(1) = 7.35, *p* = 0.022, η^2^_p_ = 0.353), ventral nucleus of ventral telencephalic area (vV) (left: X^2^_F_(1)=6.42, p=0.048, η^2^_p_ = 0.322; right: X^2^_F_(1) = 5.72, *p* = 0.048, η^2^_p_ = 0.298), ventral habenular nucleus (Hav) (left: X^2^_F_(1) = 6.06, p = 0.04, η^2^_p_ = 0.310; right: X^2^_F_(1) = 10.28, *p* = 0.012, η^2^_p _= 0.432) and ventral medial thalamic nucleus (VM) (left: X^2^_F_(1) = 6.20, *p* = 0.038, η^2^_p_ = 0.315; right: X^2^_F_(1) = 7.46, *p* = 0.011, η^2^_p_ = 0.356) (Supplementary Data 2; Fig. 2a–h). On the other hand, the left dorsal habenular nucleus (Had_l_) and the right anterior tubercular nucleus (ATN_r_) were differentially activated during asocial learning (X^2^_F_(1) = 6.86, *p* = 0.05, η^2^_p_ = 0.337 and X^2^_F_(1) = 8.42, *p* = 0.028, η^2^_p_ = 0.384, respectively; Supplementary Data 2; Fig. 2i–l).

**Table 1:** Nomenclature of brain regions and their list of abbreviations used in the present work.

Brain regions	Abbreviations
Olfactory bulbs	OB
Dorsal Telencephalic area	D
Dorsal nucleus of ventral telencephalic area	Vd
Central nucleus of ventral telencephalic area	Vc
Ventral nucleus of ventral telencephalic area	Vv
Lateral nucleus of ventral telencephalic area	Vl
Central zone of dorsal telencephalic area	Dc
Lateral zone of dorsal telencephalic area	Dl
Medial zone of dorsal telencephalic area	Dm
Posterior zone of dorsal telencephalic area	Dp
Supracommissural nucleus of ventral telencephalic area	Vs
Dorsal zone of dorsal telencephalic area	Dd
Anterior part of parvocellular preoptic nucleus	Ppa
Postcommissural nucleus of ventral telencephalic area	Vp
Magnocellular preoptic nucleus	PM
Posterior part of parvocellular preoptic nucleus	PPp
Dorsal habenular nucleus	Had
Ventral habenular nucleus	Hav
Anterior thalamic nucleus	A
Ventromedial thalamic nucleus	VM
Ventrolateral thalamic nucleus	VL
Ventral zone of periventricular hypothalamus	Hv
Anterior tuberal nucleus	ATN
Lateral hypothalamic nucleus	LH
Dorsal zone of periventricular hypothalamus	Hd
Central posterior thalamic nucleus	CP
Periventricular nucleus of posterior tuberculum	TPp
Periventricular gray zone of optic tectum	PGZ
Caudal zone of periventricular hypothalamus	Hc
Diffuse nucleus of the inferior lobe	DIL
Dorsal tegmental nucleus	DTN
Central nucleus of the inferior lobe	CIL
Nucleus lateralis valvulae	NLV
Griseum central	GC
Corpus mamillare	CM

The letters l and r were added as subscripts to identify the left and right hemispheres.

In summary, despite the behavioral similarities between social and asocial learning in zebrafish described in the previous section, learning from a social CS is associated with the local activation of different brain regions when compared to learning from an asocial CS.

### Brain functional networks associated with social and asocial classic conditioning in zebrafish

Using the correlation matrices of activity levels across the studied brain regions (i.e. matrices of co-activation), we constructed weighted networks (see methods below for details) describing the patterns of regional coactivations for each of the four combinations of experimental conditions: asocial learning (AL); social learning (SL); asocial control (AC); and social control (SC).

**Fig. 3:**
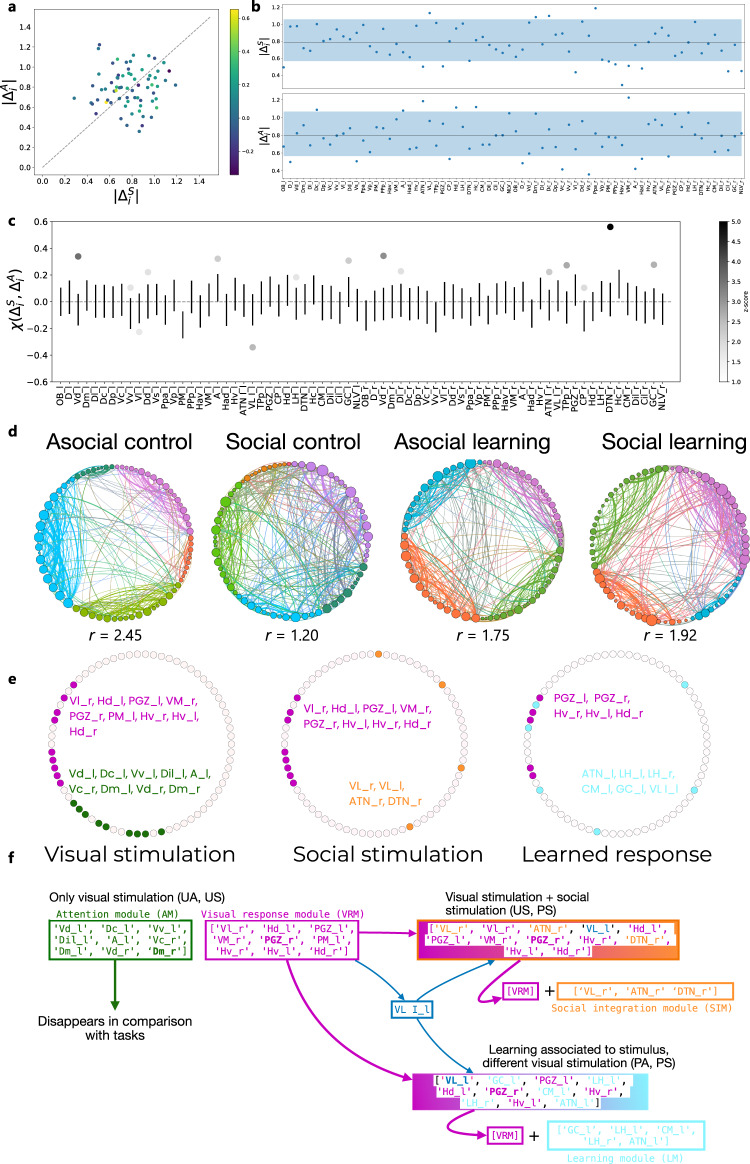
Brain networks associated with social and asocial learning. **a** Similarity of region neighborhoods between social |Δ^S^_i_| and asocial learning |Δ^A^_i_|, where the color of each data point identifies _X_(Δ^S^_i_, Δ^A^_i_). **b** |Δ^S^_i_| and |Δ^A^_i_| for all brain regions; the blue color band identifies the interval between the 5^th^ and 95^th^ percentiles of the randomized distribution; For details on the statistical significance see Supplementary Tables 1 and 2. **c** Similarity _X_(Δ^S^_i_, Δ^A^_i_) values for all brain regions. Error bars represent one standard deviation over and below the null mean value; regions marked with dots are those with ξ values significantly different from random; the gray scale encodes the z-score of the region’s = ξ value with respect to the random null model; For details on the statistical significance see Supplementary Table 3. **d** Detection of robust modules (aka communities) in the brain networks for each treatment; within each treatment, network nodes were ordered and colored according to the community they belong to, and the degree of integration (lower r) or segregation (higher r) of the networks is provided; in all cases the measured r values are significantly larger than expected (for statistical details see Supplementary Fig. 3 and Supplementary Table 4), and there are differences in integration across treatments (for statistical details see Supplementary Fig. 4 and Supplementary Table 5). **e** Conserved brain network submodules between treatments reveal a default mode network (green module), a visual response module (purple module) a social integration module (orange module) and a learning module (blue module); regions indicated in bold font are those highlighted by the analysis based on their egonetworks. **f** Schematic representation of how the identified modules are recruited to the different tasks: the common modules present in the non-learning (i.e. unpaired US-CS) treatments (i.e. UA, US) are interpreted as a default mode network (green module; this module “Disappears in comparison with the tasks” meaning that it is only present in the two control treatments and does not show up when fish are engaged in one of the behavioral tasks) and a network responding to visual stimulation (purple module), given that the latter is also present across all four conditions; the common modules between unpaired and paired US-CS (i.e. US, PS) treatments are interpreted as a social module (blue module), since the commonality between these two treatments is the presence of a social stimulus; the common module between the two learning treatments (i.e. PA, PS) is interpreted as a learning module (blue module), since the commonality between these two treatments is the US-CS pairing during the training trials.

First, we studied how regions differently integrate during social (S) and asocial (A) learning by analyzing changes in their local network neighborhoods. This was achieved by considering the difference matrices between the learning and the control treatments in response to exposure to the social and the asocial conditions. More precisely, we computed the matrix ∆^S^ = A^S,L^−A^S,C^, which quantifies the changes between learning and control in the social condition, and ∆^A^ = A^A,L^−A^A,C^, which instead quantifies changes between learning and control in the asocial condition. We found no overall significant correlation between the overall restructuring of the regional neighborhoods, quantified by module of the vector of regional differences between the two conditions (i.e. the rows ∣∆^S^_i_∣ and ∣∆^A^_i_∣ corresponding to changes to the neighborhood of region i; Fig. 3a). However, we identified a set of regions that display significant changes in activity with respect to a random null model (Fig. 3b). More in detail, a significantly (≥ 95%) large amplitude of the regional changes was found for V LI_l_, Dm_r_, Dc_r_, and Ppa_r_ in social learning and for Dc_l_, A_l_, ATN_l_, Hd_l_, Hc_l_, and VM_r_ in asocial learning, and a significantly small (< 5%) amplitude was found for OB_l_, ATN_l_, PGZ_l_, Vl_r_, PM_r_, PPp_r_, Hav_r_, VM_r_, Had_r_, Cil_r_, NLV_r_, for social and for D_l_, Vs_l_, CP_l_, D_r_, Vc_r_, Vs_r_, A_r_, Had_r_ in asocial learning. The significance was constructed by repeatedly sampling N values uniformly at random from the ∆ matrices to form a principled expectation for the amplitudes (Fig. 3b). We consider then the changes in the structure of the regional neighborhoods by computing the cosine similarity χ values between the rows corresponding to the same region of the ∆^S^ and ∆^A^ matrices (Fig. 3c). We find that the majority of regions do not display significant changes with respect to a randomized version of the neighborhood networks (see Methods). However, a few regions do display statistically significant changes. In particular, we find that regions Vl_l_ and VL_l_ are significantly (*p* < 0.05, z < −1.5) dissimilar between social and asocial learning conditions. Another set of regions instead displays significant similarity between the two conditions (*p* < 0.05, z > 1.5): Vd_l_, Vv_l_, Dd_l_, A_l_, LH_l_, DTN_l_, GC_l_, Vd_r_, Dl_r_, ATN_r_, TPp_r_, CP_r_, DTN_r_, GC_r_, implying that their local integration structure is conserved between conditions.

After having identified localized regional differences across treatments, we asked whether the network structure differs at intermediate scales (mesoscales) between treatments. This kind of deviation would signal that different information integration and elaboration strategies are used for different tasks. To this aim, we detected the modular structure of functional networks for each treatment using state of the art community detection techniques (see Fig. 3d for the results of the community detection, and Methods for the robustness analysis). The first question that we can ask is whether the modular structure differs across treatments. For example, the two control (C) treatments are characterized by a slightly larger number of communities^5^, with respect to the learning (L) treatments^4^. It is more informative however to investigate to what degree the various communities are tightly linked within themselves with respect to each other. To quantitatively characterize the differences among partitions, we measured the ratio r (see Methods) of the total edge weight within a community to the total weight the edges between communities. If *r* ≥ 1, it means that communities are denser than the inter-community medium, suggesting stronger segregation of activity within the communities as opposed to the across them. We find that the treatment asocial control (AC) has the highest *r* (*r* = 2.45), followed by the social learning (SL) (*r* = 1.92), and the asocial learning (AL) (r=1.75) treatments, highlighting the presence of better defined communities and tighter segregation of the functional activity within them in these treatments (Fig. 3d). In contrast, the social control (SC) treatment displays a lower *r* value (*r* = 1.2), indicative of higher integration across communities rather than segregation within them (Fig. 3d).

In addition to the overall balance between integration and segregation, we can ask whether these functional communities—or parts of them—are conserved across different treatments. We did this by comparing communities between partitions corresponding to different treatments and looking for intersections between them (see Methods). We first compared the two control treatments (AC and SC). We found two conserved submodules (intersections of communities): one comprising Vd_l_, Dc_l_, Vv_l_, Dil_l_, A_l_, Vc_r_, Dm_l_, Vd_r_, Dm_r_ (green module in Fig. 3e), and a second one containing Vl_r_, Hd_l_, PGZ_l_, VM_r_, PGZ_r_, PM_l_, Hv_r_, Hv_l_, Hd_r_ (purple module in Fig. 3e). Interestingly, this second submodule appears with some modifications also when comparing the social and the learning treatments (SC, SL and AL, SL). Thus, we considered it to represent a visual response module. Then, we compared the two social treatments (SC and SL) to detect conserved submodules involved in social information processing. In this case, we found a single large, conserved submodule, containing the regions VL_r_, Vl_r_, ATN_r_, VL_l_, Hd_l_, PGZ_l_, VM_r_, PGZ_r_, Hv_r_, DTN_r_, Hv_l_, and Hd_r_. Note that this module contains a large part of the second module conserved between the control treatments (AC and SC), with the addition of regions VL_r_, ATN_r_, VL_l_ and DTN_r_ (i.e. social integration module = orange module in Fig. 3e, f). Finally, we compared the two learning treatments (AL and SL) to detect conserved submodules involved in general learning (i.e. retrieval of learned response in both social and asocial learning) and found a single large, conserved submodule containing regions VL_l_, GC_l_, PGZ_l_, LH_l_, Hd_l_, PGZ_r_, CM_l_, Hv_r_, LH_r_, Hv_l_, and ATN_l_. Note again that this submodule contains a large fraction of the second module found in the comparison between controls (AC-SC), with the additional regions GC_l_, LH_l_, CM_l_, LH_r_, and ATN_l_ (i.e. general learning module = blue module in Fig. 3e, f). In all cases, we found that the conserved submodules include region VL_l_.

## Discussion

In this study zebrafish learned equally well a CS-US pairing using either social or an asocial CS, as there were no significant differences between the social and asocial learning treatments nor in learning acquisition during training phase neither in retrieval during the probe test. Importantly, we confirmed that zebrafish were able to discriminate between the social and asocial cues (i.e. CSs) used in this study and, as previously described, that they have a preference for the social cue^34^. Therefore, social and asocial classic associative learning seem to be equally efficient.

When we analyzed the levels of brain activation, quantified by *c-fos* expression, we found that retrieval of social learning is associated with increased activity in the olfactory bulbs (OB), the ventral zone of ventral telencephalic area (Vv), the ventral habenular nuclei (Hav) and the ventromedial thalamic nuclei (VM), whereas retrieval of asocial learning is associated with a decrease in activity in the dorsal habenular nuclei (Had) and in the anterior tubercular nucleus (ATN). Interestingly, all the brain regions associated with social learning have been previously implicated in learning tasks. The OB has been described as an important brain region for social learning. The cryptic cells (a subtype of cells in olfactory bulbs) are recruited in kin recognition^35^, and an increase of GABA and glutamate in mitral cells is observed after training in social transmission of food preference^36–39^. In our learning paradigm the CS is a visual cue and the US can be perceived either by visual or chemical cues. Thus, the increased activity of the OB cannot be explained as a direct response to the visual CS in the probe test phase, but rather as a conditioned response to it after successful pairing of the chemical US with the CS during the training phase. Thus, the expectation of food is apparently increasing the activity of the OB in anticipation of a feeding event, suggesting a modulation of olfactory perception by the social CS. The association of Vv with social learning in zebrafish is not surprising given that the lateral septum, which is its putative homolog in mammalian brains, has been implicated in several learning processes, such as auditory fear learning^40,41^, contextual learning^40,42^, and working memory^43^. Other studies revealed the role of Vv in the processing of social information, such as social orientation^44^, audience effects^45^ and social exploration^46^. Together, this evidence is congruent with our findings, where Vv is crucial to learning related from social cues. The lateral habenula (LHb), which is the mammalian putative homolog of the Hav^47^, has also been implicated in learning and memory. For instance, inhibition of the LHb led to deficits in spatial memory^48^, object recognition^49^, spatial working memory^50^, aversive conditioning to cocaine^51^, and complex conditioning task^52^. Moreover, social behavior is also regulated by the LHb as evidenced by the decrease of *c-fos* expression in the LHb in social isolation, by the reduction in *c-fos* expression during social play^53^, and by the impair of social behaviors by the activation of LHb or pre-frontal cortex (PFC) neurons and PFC-LHb projections^54–57^. Finally, the VM, which is considered as a thalamic nuclei in zebrafish^58^, belongs to the cortico-basal ganglia-thalamic loop circuit in mammals, in which the basal ganglia receive inputs from the cortex and transfers them back to frontal and motor cortex via the VM^59^. This circuitry is also connected with LHb allowing animals to adjust the salience and valence of stimuli.

In contrast, retrieval of asocial learning in zebrafish is associated with other brain regions, namely the dorsal habenular nuclei (Had) and the anterior tubercular nucleus (ATN). The Had, which is homologous to the medial habenula in mammals (Ham), receives inputs mainly from the limbic system, and sends outputs to the interpeduncular nucleus, which in turn regulates activity dopamine (DA) and serotonin (5HT) neurons^60–62^. Evidence in both mice and zebrafish supports our results that suggest Had to be related to asocial learning. Ablation of Ham induces deficits in long-term spatial memory^54^, complex learning paradigms^61^. and fear learning^63,64^. In contrast, our findings reveal a decreased expression of *c-fos* associated with asocial learning, suggesting a decreased activity of an inhibitory circuit. The ATN is homologous to the ventromedial hypothalamus (VMH) in mammals, a brain region that has also been related to learning processes with strong *c-fos* expression after fear conditioning^65^. and recall of conditioned fear^66^. The role of VMH in learning processes in mammals can be explained by the afferents from the amygdala (BLA and MEA), a brain region clearly shown to be involved in learning processes^65^. In summary, social and asocial learning in zebrafish are associated with changes in activity in different sets of brain regions known to be involved in learning in other species.

We have also studied the structure of brain networks in relation to the retrieval of the two types of learning. The regional similarity data also reveals a lack of correlation between social and asocial learning, and a large amplitude of the change in the neighborhood structure (i.e. implying more marked local changes in network structure) for different regions in the two types of learning (i.e. social: VL_l_, Dm_r_, Dc_r_, Ppa_r_; asocial: Dc_l_, A_t_, ATN_l_, Hd_l_, Hc_l_, VMr). Furthermore, the community detection results revealed a robust modularity of the brain networks across all treatments, with the social learning treatment displaying a significantly higher integration across communities than the asocial one. Finally, we investigated the composition of shared submodules in an attempt to frame and contextualize them. We offer a hypothesis based on these results. The treatments AC-SC share the visual response to a stimulus but include no learning on the part of the individuals. We can imagine therefore that the shared submodules will encode the simple reaction of the animal to the appearance of a visual stimulus carrying no semantic meaning (as the animal has not learned to associate it with food). The two submodules should therefore code for the generalized attention (AM) and the visual response mechanisms (VRM) (Fig. 3f). In the comparison SC-SL, the commonality lies in the presence of a social visual stimulus. We would expect therefore to see a recruitment of the VRM with a potential additional recruitment of other regions responsible for social recognition. From this perspective, the single conserved submodule that we found in the SC-SL supports this interpretation, being largely composed by the VRM regions and a few additional ones, that we now denote as social integration module (SIM) (Fig. 3f). Along the same line, the AL-SL should highlight the submodule specific to learning (i.e. retrieval) and reacting to the food stimulus, independently from the type of visual stimulus. We find again a single large, conserved submodule, that includes about 50% of the VRM regions plus a new set of regions with no overlap with the SIM. Arguably, these additional regions should be responsible for the learned response and association with the food stimulus, and we denote them as the learning module (LM) (Fig. 3f). Finally, the region VL_l_ constitutes a glaring exception, as it appears in all the submodules that we described so far. This general presence might suggest that it has a generic role in information integration across different areas.

It should also be noted that, although there was very little laterality in the level of activity of the nodes of the network (as indicated by similar levels of activity between the left and the right side in most brain regions that express differences in activity associated with the retrieval of the learned response), there was a strong lateralization in the functional connectivity across the network, such that only a handful of brain regions are bilaterally represented in each of the functional modules described above (i.e. a large number of brain regions are represented in the modules only by the left or right side). Functional brain lateralization of different cognitive modules (e.g. perception/action, emotion, and decision-making, e.g. ^67^) has been described for humans and brain asymmetries and their behavioral effects have been described throughout the animal kingdom^68,69^. Given the eye-brain contralateral projections present in vertebrates, lateralization of visual behaviors has been used to study brain lateralization, from fish to mammals. Visual lateralization has been found in different aspects of social behavior, with most fish species tested so far, including zebrafish, expressing a left eye - right hemisphere preference for inspection of social stimuli^70–72^. In agreement with this behavioral lateralization, in our study, the social module (SIM in Fig. 3f) is exclusively composed by brain regions present in the right hemisphere. Similarly, the learning module (LM in Fig.3f) is also lateralized, but in this case, it is mostly composed by left-hemisphere structures. These results suggest the occurrence of laterality in zebrafish brain function, that may increase efficiency in information processing by avoiding duplication of functions in the two hemispheres, hence increasing neural capacity^68,69^.

In summary, here we show that social and asocial learning are associated with localized differences in brain activity that are paralleled by the segregation of brain modules that seem to serve subsets of cognitive functions, such as a visual response module, a social integration module and a learning (i.e. retrieval) module that is shared between the two types of learning. Together, our results provide evidence for the occurrence of a general-purpose learning module that is apparently modulated by different patterns of localized activity in social and asocial learning.

## Methods

### Animals

Zebrafish (*Danio rerio*) were 5 months old wild-type (Tuebingen strain) males, bred and held at Instituto Gulbenkian de Ciência Fish Facility (Oeiras, Portugal). Fish were kept in mixed-sex groups, at 28 °C, 750 µS, pH 7.0 pH in a 14L:10D photoperiod and fed twice a day (except on the day of the experiments) with freshly hatched *Artemia salina* and commercial food flakes.

### Ethics statement

All experiments were performed in accordance with the relevant guidelines and regulations, reviewed by the Instituto Gulbenkian de Ciência Ethics committee, and approved by the competent Portuguese authority (Direção Geral de Alimentação e Veterinária, permit number 0421/000/000/2017).

### Behavioral paradigm

One day before the experiment, fish were moved to the home tanks (1.5L, 12.5 cm × 12.5 cm × 12.5 cm) where they only had visual and chemical access to a mix shoal of 4 animals (2 familiar males and 2 familiar females).

The experiment was subdivided into three phases: acclimatization, training and probe test. In the acclimatization phase, after one minute in the start box, animals were allowed to swim freely in the tank for 9 min, during which, they were attracted to all arms of the plus-maze with bloodworms, so that they became familiar with the whole maze. In the training phase, animals were trained in daily sessions of 8 sucessive trials (2 min each) per session for 6 days. In the paired groups, animals had the CS and the US presented together in the same arm, and received a reward (bloodworm) when this arm was chosen (that changed on each trial in a pseudo-randomized way, within and between individuals); when another arm was chosen, animals stayed one minute in the chosen arm, and then they were conducted to the right arm, using a hand net where they receive the reward. In the unpaired groups, the animal spent 2 min in the chosen arm since the CS and US were never presented together. In both groups, when individuals reached the RoI of the chosen arm the start box was closed to prevent the animal changing its decision. In the social treatments, the CS stimulus presented at the end of the arm was a static, 2D photograph print-out of a zebrafish. In the asocial treatments a digitally drawn circle print-out with the same visual target area and the same mean color of the zebrafish-stimulus was used. After each training session, individuals returned to their home tank. In the probe test (24h after the last training trial), animals were only exposed to the CS for 2 min The CS was then removed and the animal remained in the tank for 30 min to achieve the peak of expression for *c-fos*^73^.

A learning discrimination test was used to assess if fish can discriminate between the fish and the circle stimuli. For this purpose, in a rectangular tank, we presented one of the stimuli (same print-out of the social or asocial stimuli described above) in one end and the other in the other end, and in a series of training trials we have paired one of the stimuli with a food reward. Each fish was randomly assigned to training trials to reward either the social or the asocial stimuli. Then, in a probe trial we have tested the choice of fish between the two stimuli to check if they have developed a preference for the rewarded stimulus, which would indicate the ability to discriminate between the two used stimuli (fish vs. circle).

A preference test was performed to assess if individuals prefer social to asocial CS’s. For this purpose, we used a rectangular tank (5L, 30 cm × 15 cm × 15 cm) with the stimuli presented on each side (e.g. social stimulus in the left and asocial stimulus in the right side, in a randomized way between individuals; same print-outs as described above were used). Individuals were placed in a start box for 2 min with transparent partition, and the cumulative time spent in both RoI’s was compared.

To test if the association of a food reward with either the circle or the fish shape is specific for each cue, we trained wild-type zebrafish in a simple reward learning paradigm. Individuals were introduced to a central compartment of a rectangular arena (29.5 × 14.5 × 11 cm), constructed by removable transparent dividers, and acclimatized to ambient conditions that included the projection of a white background from computer monitors set at each end of the arena (Asus LCD, VG248, Full HD1080, 144 Hz refresh rate). Following acclimation, at each trial animals were presented with either the circle or fish shape (same images as those used in the other experiments) against a white background on one monitor, while the empty white background remained on the other. At this point, the dividers were lifted so that animals could approach either monitor, followed by replacement of the dividers, but only approach towards the side of the cue was instantly rewarded by a food reward—a single bloodworm released by pipette. Following receipt of the food reward, the projection of the shape was removed from the screen and the single divider for that side was lifted to allow the animal access back to the central compartment. After entering the compartment, the divider was replaced, and animals were given a 1 min interval before repeating the trial again. Based on learning plateaux thresholds established by previous work on similar learning paradigms in zebrafish^74,75^, a total of 16 trials were performed. These were separated in two blocks of 8 trials over 2 consecutive days and the side in which the rewarded cue was presented was counterbalanced between experimental animals. Learning was indicated by animals reaching preference for the cue location, over the no-cue location or the no-choice option, on day 2. As such, learning criterion was set at a success rate that statistically exceeded chance level between the three options, at ≥ 5/8 trials (1-sample binomial test: p̂ > 1/3; *Z* = 1.75, *P* < 0.05). Overall, 50 animals were trained, out of which 33 reached learning criterion, 17 trained with the circle and 16 with the fish shape, which amounts to a 66% learning success in the sample population. Following training, animals that learned were exposed to a single non-rewarded probe trial 24 h after their last training trial. This trial included the same acclimation procedure, but fish from either the circle or the fish-shape learning groups were tested with either a congruent cue (circle-trained tested with the circle or fish-trained tested with the fish) or an incongruent cue (circle-trained tested with or fish-trained tested with the circle). This trial tested for the specificity of each cue in associative learning, as opposed to potential generalization across cues, by comparing success rates between congruent and incongruent cue tests for both circle-trained and fish-trained animals.

In all experiments, the behavior was recorded with a digital camera for subsequent analysis using commercial video tracking software (EthoVisionXT 8.0, Noldus Inc. the Netherlands). The extracted behavioral measures are included in Supplementary Data 1.

### Brain collection

Animals were sacrificed with an overdose of Tricaine solution (MS222, Pharmaq; 500–1000 mg/L) and sectioning of the spinal cord. The brain was macrodissected under a stereoscope (Zeiss; Stemi 2000) and immediately collected to 4% PFA solution in 0.1M phosphate buffer (PB) and kept overnight at 4 °C. After cryopreservation (34% sucrose in 0.1M PB ON at 4 °C), the brains were embedded in mounting media (OCT, Tissue tek) and rapidly frozen on liquid nitrogen. The coronal sectioning was performed on a cryostat (Leica, CM 3050S) at 16 um, sections were collected onto SuperFrost glass slides and stored at −20 °C.

### In situ hybridization for the immediate early gene *c-fos*

Chromogenic RNA in situ hybridization (CISH) was carried out according to a standard protocol available upon request from the lab of Professor Marysia Placzek, University of Sheffield, briefly described below. For the generation of *c-fos* probes, a pBK-CMV vector containing the *c-fos* cDNA (Genebank: CF943701) was cut with the restriction enzyme BamHI (antisense) and EcoRI (sense) to generate templates for in vitro transcription. Digoxigenin-labeled *c-fos* sense and antisense probes (11277073910, Merck (Roche), UK) were then synthesized through in vitro transcription of 1 mg template with T7 polymerases (M0251, New England Biolabs). The sections were fixated in 4% PFA, washed in PBS, rinsed in 0.25% acetic anhydride in 0.1 M tri-ethanolamine for 10 min and washed 3 times in phosphate buffer saline (PBS). An incubation in pre-hybridization buffer (hybridization solution without yeast RNA, minimum 3 h) was done in order to prepare the tissue for receiving the probe diluted in hybridization solution (probe dilution: 1:40 ~4 ng/ul final concentration). The hybridization buffer contained 50% formamide, 5 × SSC (pH 7.0), 2% blocking powder, 0.1% triton X-100, 0.5% CHAPS, 1 mg/ml yeast RNA, 5mM EDTA, and 50 ug/ml heparin. The hybridization incubation was performed at 68 °C for 24 h. Following hybridization, the sections were treated with secondary antibody anti-dig-AP (1:1000, 11093274910, Merck (Roche), UK), after a series of several washes decreasing concentrations of SSC, until 0.1× SSC. The tissue was then mounted onto GlicerolGel (GG1, Merk) coated slides and left to air dry.

### Cell counting

The slides were imaged using a tissue scanner (NanoZoomer Digital Pathology, Hamamatsu). A whole brain screening was performed to select the brain nuclei with higher *c-fos* activity to be counted (see list in Table1). The brain regions of interest were delimited based on the available zebrafish adult brain atlas^76^ and on a series of DAPI-stained sections across the whole brain available in the lab for visual guidance. The areas were manually drawn, and the signal was automatically quantified using the Icy software (created by the Quantitative image analysis unit at Institut Pasteur). The sum of the cross sections was used as an individual measure for each area side.

### Statistics and reproducibility

Experimental groups size was kept between 9 and 30 animals, which was determined as the minimum necessary to identify differences with higher effect sizes at a statistically significant confidence levels (*p* > 0.05), for reducing false-report probability^77^ and simultaneously reducing the animal use required when considering rates of exclusion based on learning and retention abilities during training.

A chi-square test was used to compare proportions of learners, non-learners and non-retention individuals, relative to the total amount of individuals in each treatment, between social and asocial treatments, with the odds ratio (OR) used to calculate effect size based on the rate of proportion differences. Non-parametric linear regressions were performed to compare the learning curves across the 4 experimental treatments. To assess differences between the experimental treatments during the probe trial, a non-parametric test on cumulative duration was conducted with the location in the plus maze (target arm, front, left or right arms) as within factor and social (social, asocial) and learning (learners, non-learners) factors as between factors. Effect size was measured by the partial eta-squared value (η p 2ηη^2^_p_), calculated via :

1 $${{\eta }^{2}}_{p}=\frac{F\times {{df}}_{n}}{F\times {{df}}_{n}+{{df}}_{d}}$$

For the two-choice conditioning experiment used to test discrimination learning, non-parametric linear regressions were used to compare learning curved between animals trained with the asocial cue (circle) and those trained with the social cue (fish shape), with η^2^_p_ as a measure for effect sizes. For the local interaction test used to examine cue preference, we compared the proportion time in the region near each cue using a paired t-test, again with η^2^_p_ as an effect size estimate. For the two-choice appetitive discrimination-learning experiment, used to test the specificity of each cue (circle or fish shape) in associative learning, we first tested if success rates (i.e. proportion correct choice) differed across training trials via a binary logistic regression with a logit function. For each training trial, we also use one-sample binomial tests to examine if average success rates significantly exceeded the 50% level of no preference, to identify learning of the correct choice. Comparisons of average success rates between training blocks were also conducted via two-sample binomial tests. Finally, two-sample binomial tests were also used to compare success rates between animals tested with a congruent cue and those tested with an incongruent cue during probe trials, with effects denoted by the difference in success proportion (Δp̂). Effect sizes were measured by the OR, either by congruent relative to incongruent treatments, or for each treatment compared to chance choice (50%).

The effect of social learning on brain activity in the probe test was assessed by a non-parametric test with laterality (number of *c-fos* positive cells on each nuclei on the left and right side of each brain) as repeated measure and social and learning as between factors area-by-area (OB, D, Vd, Vc, Vv, Vl, Dc, Dl, Dm, Dp, Vs, Dd, PPa, Vp, PM, PPp, Had, Hav, A, VM, VL, Hv, ATN, LH, Hd, CP, TPp, PGZ, Hc, DIL, CIL, DTN, NLV GC, CM). Planned comparisons followed by Benjamini and Hochberg’s method for p-value adjustment were used across all annotated brain regions, to assess the brain areas associated with social learning (social learning vs social control) and asocial learning (asocial learning vs asocial control). All statistical procedures described above were run on GraphPad-Prism v. 9.4.

### Network analysis

Functional networks for brain connectivity are usually built starting from vectorial information on the individual regions. In network neuroscience, this means computing a similarity measure (e.g. Pearson correlation) between the timeseries of brain parcels in fMRI, or channels in EEG. In the case of social learning experiments the correlations of number of positive cells for each pair of brain nuclei, for within each experimental treatment was computed, however we have access to a single measure per region for each specimen. In addition, the number of specimens is typically limited. In turn this makes the estimation of the actual correlation (or similarity) between regions more complicated.

To account for this, we take inspiration from standard bootstrapping and, instead of defining a single network, we construct a set of possible networks leaving out some of the specimen information’s.

More precisely, consider the case of $M$ specimens, each with one sample reading ${x}_{i}$ for each of the $N$ brain regions. Given a similarity metric $\omega$, typically one would consider the $M$-dimensional vectors ${x}_{i}$, where $i$ labels the regions, and then compute the similarities

2 $${\omega }_{{ij}}=\omega ({x}_{i},{x}_{j})$$

for all pairs ${ij}$. The resulting weighted matrix $\varOmega$ is then interpreted as the adjacency matrix of a functional network.

For a given $m < M$, we will instead consider all the $\left(\frac{M}{m}\right)$ combinations $\{\gamma \}$ of $m$ specimens and compute the corresponding functional graph ${\varOmega }_{\gamma }$. We will refer to the collection of graphs obtained in this way as a graph tower ${\varOmega }_{\varGamma }$, where each of the combinations can be considered as a graph layer. Similar constructions are used for multilayer and multiplex networks with the notable difference that graph towers do not have edges connecting the different layers.

The advantage of this construction is that each layer in the graph tower represents a different instance of the network bootstrapping. In this way, observables computable on a single layer can be bootstrapped across multiple ones. This construction has naturally one parameter, the sampling number m, which needs to be chosen on the basis of data-driven considerations or the robustness of the resulting networks.

Correlation networks are usually fully connected weighted networks. It is however common to sparsify them by retaining only edges that have a weight larger than a certain weight threshold. Another common practice is to choose a target density ρ for the graph and add edges to the network starting from the strongest ones until the density is reached. Given a graph (layer) Ωγ, we will denote the graph obtained using a threshold Ωγ at density ρ as Ω^ρ^_γ_. While the sparsification is often required to highlight the network properties of the system and to filter out weaker correlations, there is no commonly accepted method to choose such thresholds. Typically, the adopted methods depend strongly on the specific application and are developed ad-hoc. Overall, most existing methods rely either on considerations on the data used to construct the correlation matrix (e.g. the timeseries in neuroimaging), or on the local structure of the network (e.g. disparity filter).

Here, we take a different route and leverage the graph tower structure to choose the threshold value. We will work using the density as threshold, but the same argument can be replicated using weights in a straight-forward manner. For each edge ij, we can consider the set of edge weights {ω_ij_}_γ_ across all layers {γ}. Denoting respectively µ(ω_ij_) and σ(ω_ij_) as the mean and standard deviation of the ω_ij_ over the layers, we can associate to Ω a mean heterogeneity

3 $$\zeta ({{\Omega }})={{\langle }}\sigma (\omega {ij}){{\rangle }}({ij})$$

and a mean coefficient of variation

4 $$\chi (\Omega )=(\frac{\sigma \left(\omega {ij}\right)}{\mu \left(\omega {ij}\right)})\,({ij})$$

Denoting as Ω^ρ^_Γ_ the graph tower thresholded at density ρ, the two quantities above can be computed as a function of the threshold density ρ. In Supplementary Fig. 2a, b we report the dependence of ζ and χ. We find a clear change in the heterogeneity patterns at around ρ_0_ = 0.05. In particular, near ρ_0_ the mean heterogeneity ζ is still minimal, while the coefficient of variation χ peaks before starting to decrease again. In Supplementary Fig. 2c we report for comparison a recent method for density thresholding proposed in^78^. This method identifies ρ_0_ as the density that maximizes the quantity

5 $${J}=({Eg}+{El})/p$$

where Eg and El are respectively the global and local network efficiency^79^. Interestingly, we find the threshold density identified by J is very close to the ρ_0_ identified by ζ and χ, suggesting that our construction based on the heterogeneity patterns of Ω_Γ_ captures a critical point in network structure. In the rest of the paper, all the networks will be thresholded at this density ρ_0_.

We are interested in comparing how brain regions are linked to each other in the various conditions and tasks. During different tasks, the same brain region might be performing different functions by changing how it links to the other regions, its local context (also called egonetwork). Given two conditions A and B with associated matrices A^A^ and A^B^, we can measure then the change in the local environment of a region i by computing the amplitude and direction of the change. The amplitude of the change can be quantified by considering the norm of the difference between the row vectors associated to i in the two conditions

6 $$AiA=(Ai0,\,.\,.\,.\,A{iN}-1)$$

and

7 $$AiB=\left(A\,Ai0,\,.\,.\,.\,A\,B{iN}-1\right).$$

That is, we calculated the vector difference between the two rows

8 $$\triangle {AB}i=| AAi-ABi|$$

and then take its norm ∣∆^AB^_i_ ∣. To quantify the direction of the change, we can instead the cosine similarity between A^A^_i_ and A^B^_i_ :

9 $${\chi }_{i}\left(A,B\right) = \frac{\mathop{\sum}\limits_{j}{A}_{{ij}}^{A}{A}_{{ij}}^{B}}{\sqrt{\mathop{\sum}\limits_{j}{({A}_{{ij}}^{A})}^{2}\sqrt{\mathop{\sum}\limits_{j}{({A}_{{ij}}^{B})}^{2}}}}$$

Communities were computed using the Leiden community detection method^80^ on the graph tower matrices, averaged over all the bootstrapping samples at fixed density *p* = 0.07. To increase the robustness of the detection, for each treatment, we repeated the community detection 100 times. From the 100 candidates partitions we extracted the central partition as described in^81^ and associated the resulting partition to the treatment under analysis.

To quantitatively characterize differences among partitions, we measure the ratio *r* of total edge weight within a community with that of the edges between communities. More specifically, for partition P with m communities we compute the (*m* x *m*) matrix P, defined as:

10 $$P\alpha \beta ={\sum }_{i\in a,j\in \beta }\omega {ij}$$

Where

11 $$\alpha ,\,\beta =0,\,\ldots m-1$$

label the modules of *P*, and *ωij* is the edge weight between regions I and j.

We then compute the ratio of average intra-community edge weights as follows:

12 $$r=\frac{(m-1)\,\sum \, \alpha \,{Paa}}{2 \, \sum \, \ne \, \beta \, P \, \,\alpha \,\beta }=\frac{(m-1)}{2}\,\frac{{TrP}}{\left|\left|P\right|\right|1-{TrP}}$$

Which measures the ratio of the average weight on the diagonal of P_α, β_ to the average off-diagonal weight.

We would also like to identify modules, or parts of modules, that are shared across partitions corresponding to different treatments. One method to quantify this is to study the overlap between pairs of modules: consider partitions

13 $${Px}=\{C0x,{C}1x\,.\,.\,.{C}mx\}{and\,P}y==\{C0y,{C}1y...Cly\}$$

for treatments x and y; for each pair of modules (C^i^_x_, C^j^_y_), we compute the intersection

14 $$\,Ji,jx,y=Cix\cap Cjy.$$

To establish significance, we employ a permutation test based on a null distribution for the size of intersections p_0_(∣J∣): for each pair (C^i^_x_, C^j^_y_), we sample uniformly at random 10000 pairs of node sets with cardinality respectively ∣C^i^_x_∣ and ∣C^j^_y_∣ and compute the size of their intersection ∣J∣. We then retain the submodule

15 $$J^{i,\,j}x,\,y^{iff}| J^{i,\,j}x,y| \,\ge \,\mu \,(| J| )+3\sigma \,(| J| )$$

where μ(∣J∣) and σ(∣J∣) are the first two moments of p_0_(∣J∣).

### Reporting summary

Further information on research design is available in the Nature Portfolio Reporting Summary linked to this article.

## Supplementary information

Supplementary Information

Description of Additional Supplementary Files

Supplementary Data 1

Supplementary Data 2

Reporting Summary

## Data Availability

The datasets used in the current study have been submitted as supplementary files, with source data for Figs. 1 and 2 found in Supplementary Data 1 (grouped by experiment). Source data for Fig. 3 found in Supplementary Tables 1 – 5.

## Code availability

The code for the network analysis is available at https://github.com/nplresearch/social-asocial-learning and at Zenodo with10.5281/zenodo.7954376^82^.
