# Supplementary material for: Social and asocial learning in zebrafish are encoded by a shared brain network that is differentially modulated by local activation
Source: Commun Biol. 2023 Jun 13;6:633. doi: 10.1038/s42003-023-04999-5 (PMC10260970; doi:10.1038/s42003-023-04999-5)
Supplement: Supplementary file 1 — Supplementary Information [file 42003_2023_4999_MOESM1_ESM.pdf]

Supplementary materials for

Social and asocial learning in zebrafish are encoded by a shared brain network that is differentially modulated by local activation

Júlia Pinho, Vincent Cunliffe, Kyriacos Kareklas , Giovanni Petri, Rui F. Oliveira*

* corresponding author. E-mail: ruiol@ispa.pt

This PDF file includes:
Supplementary Tables 1 to 5
Supplementary Figures 1 to 4

Supplementary Tables

Supplementary Table 1. P-values for regions with significantly similar neighbourhoods between treatments S and A.

Region	S	A
VL_l	0.0241	-
Dm_r	0.0379	-
Dc_r	0.0337	-
Ppa_r	0.0132	-
Dc_l	-	0.0365
A_l	-	0.0408
ATN_l	-	0.0092
Hd_l	-	0.027
Hc_l	-	0.0242
VM_r	-	0.0049

Supplementary Table 2. P-values for regions with significantly dissimilar neighborhoods between treatments S and A.

	S	A
OB_l	0.0096	-
ATN_l	0.0123	-
PGZ_l	0.0136	-
Vl_r	0.0018	-
PPp_r	0.0247	-
Hav_r	0.0	-
VM_r	0.0163	-
Had_r	0.007	0.0283
Cil_r	0.0027	-
NLV_r	0.003	-
D_l	-	0.013
Vs_l	-	0.016
CP_l	-	0.0272
D_r	-	0.0089
Vc_r	-	0.0008
Vs_r	-	0.0001
A_r	-	0.0011
TPp_r	-	0.0477

Supplementary Table 3. P-values and z-scores for significant similarity values $\chi(\Delta^S_i,\Delta^A_i)$. Negative z-scores correspond to regions with significantly smaller similarity between S and A, implying that their neighborhood changes strongly. Positive z-scores correspond to regions whose neighborhood is significantly conserved between S and A.

region	p-value	z-score
Vl_l	0.0406	-1.6740
VL_l	0.0174	-2.6605
PGZ_r	0.0452	-1.5724
Vd_l	0.0022	3.5444
Vv_l	0.0256	1.8217
Dd_l	0.0330	1.8269
A_l	0.0136	2.2737
LH_l	0.0420	1.7638
DTN_l	0.0008	5.2837
GC_l	0.0286	2.1504
Vd_r	0.0016	3.0605
Dl_r	0.0426	1.8000
ATN_r	0.0130	2.1963
TPp_r	0.0028	2.8309
CP_r	0.0152	2.0047
DTN_r	0.0004	4.6502
GC_r	0.0146	2.5413

Supplementary Table 4. *r values with significance.*

treatment	r	p-value
(P, A)	1.750281	1.0000
(P, S)	1.926310	1.0000
(U, A)	2.522384	1.0000
(U, S)	1.203354	0.9995

Supplementary Table 5. *Δr values with significance.*

treat 1	treat 2	$\Delta r = r_1 - r_2$	p-value
(P, A)	(P, S)	-0.176029	0.0485
(P, A)	(U, A)	-0.701090	0.0000
(P, A)	(U, S)	0.656638	1.0000
(P, S)	(U, A)	-0.525061	0.0000
(P, S)	(U, S)	0.832667	1.0000
(U, A)	(U, S)	1.357728	1.0000

Supplementary Figures

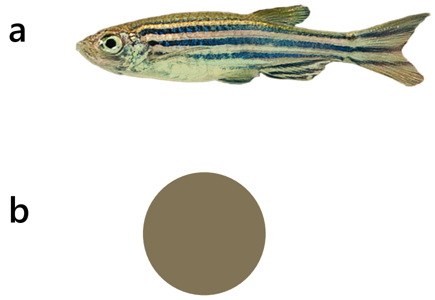

Supplementary Figure 1. Static visual stimuli used in the experiment. These include (a) a static picture of a fish social stimulus; (b) and an image of a circle, as an asocial stimulus.

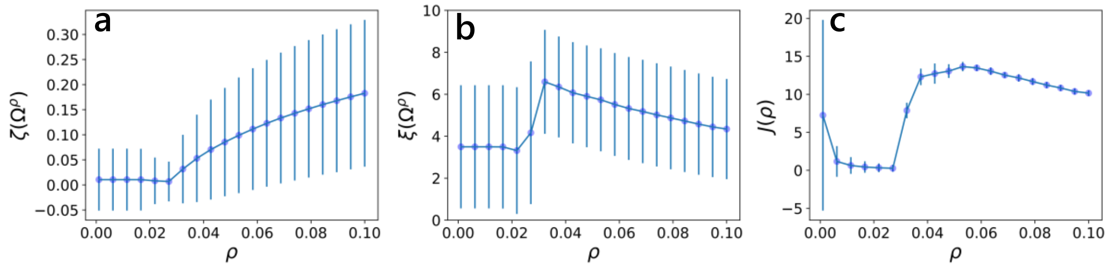

Supplementary Figure 2. Heterogeneity and efficiency of graph tower as function of density ρ.

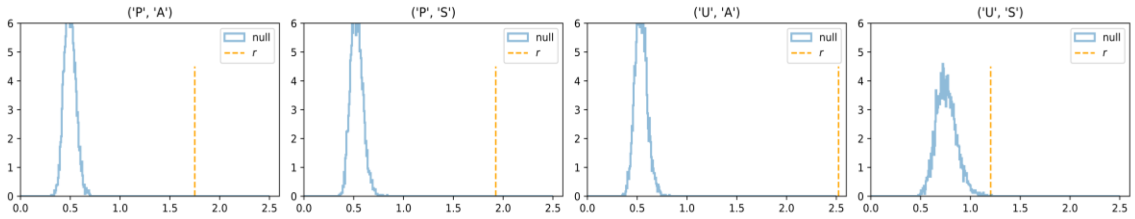

Supplementary Figure 3. Null distributions for r values. The panels show the null distributions p(r') (blue) and the r values obtained from data for each treatment. In all cases, the measured r are (very significantly) larger than expected from the null model.

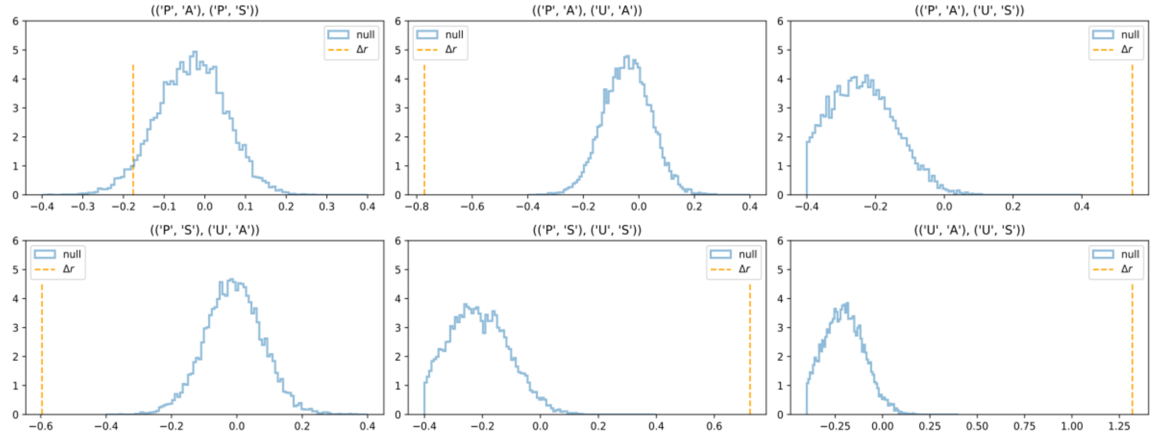

Supplementary Figure 4. Null distributions for Δr values. The panels show the null distributions p(Δr') (blue) and the Δr values obtained from data for each pair of treatments. The measured Δr are very significantly different from the null expectation, with the exception of the comparison PA-PS which marginally significant for the significance threshold α=0.05.
