# Supplementary material for: Social and asocial learning in zebrafish are encoded by a shared brain network that is differentially modulated by local activation
Source: Commun Biol. 2023 Jun 13;6:633. doi: 10.1038/s42003-023-04999-5 (PMC10260970; doi:10.1038/s42003-023-04999-5)
Supplement: Supplementary file 2 — Description of Additional Supplementary Files [file 42003_2023_4999_MOESM2_ESM.docx]

**Description of Additional Supplementary Files**

**File name:** Supplementary Data 1

**Description:** Source data behind the graphs in the paper.

**File name:** Supplementary Data 2

**Description:** Statistical calculation outputs and effect-size measures for local activation analyses for all brain regions.
